# PhenoVar: a phenotype-driven approach in clinical genomics for the diagnosis of polymalformative syndromes

**DOI:** 10.1186/1755-8794-7-22

**Published:** 2014-05-12

**Authors:** Yannis J Trakadis, Caroline Buote, Jean-François Therriault, Pierre-Étienne Jacques, Hugo Larochelle, Sébastien Lévesque

**Affiliations:** 1 Department of Medical Genetics, McGill University Health Centre, Montreal, Canada; 2 Department of Paediatrics, division of medical genetics, Faculty of Medicine and Health Sciences, Université de Sherbrooke, Sherbrooke, Canada; 3 Department of Computer Science, Faculté des Sciences, Université de Sherbrooke, Sherbrooke, Canada; 4 Département de Biologie, Faculté des Sciences, Université de Sherbrooke, Sherbrooke, Canada; 5 Medical geneticist, Biochemical Genetics Fellow, McGill University Health Centre, The Montreal Children’s Hospital, 2300 Tupper Street, Room A-604, Montreal H3H 1P3, Qc, Canada; 6 Medical geneticist, Department of Paediatrics, Medical director molecular genetics laboratory, Centre Hospitalier Universitaire de Sherbrooke, Sherbrooke, Canada

**Keywords:** Genome, Exome, Encrypted, Sequencing, Clinic, PhenoVar, I-MPOS, I-MPOSE

## Abstract

**Background:**

We propose a phenotype-driven analysis of encrypted exome data to facilitate the widespread implementation of exome sequencing as a clinical genetic screening test.

Twenty test-patients with varied syndromes were selected from the literature. For each patient, the mutation, phenotypic data, and genetic diagnosis were available. Next, control exome-files, each modified to include one of these twenty mutations, were assigned to the corresponding test-patients. These data were used by a geneticist blinded to the diagnoses to test the efficiency of our software, PhenoVar. The score assigned by PhenoVar to any genetic diagnosis listed in OMIM (Online Mendelian Inheritance in Man) took into consideration both the patient’s phenotype and all variations present in the corresponding exome. The physician did not have access to the individual mutations. PhenoVar filtered the search using a cut-off phenotypic match threshold to prevent undesired discovery of incidental findings and ranked the OMIM entries according to diagnostic score.

**Results:**

When assigning the same weight to all variants in the exome, PhenoVar predicted the correct diagnosis in 10/20 patients, while in 15/20 the correct diagnosis was among the 4 highest ranked diagnoses. When assigning a higher weight to variants known, or bioinformatically predicted, to cause disease, PhenoVar’s yield increased to 14/20 (18/20 in top 4). No incidental findings were identified using our cut-off phenotypic threshold.

**Conclusion:**

The phenotype-driven approach described could render widespread use of ES more practical, ethical and clinically useful. The implications about novel disease identification, advancement of complex diseases and personalized medicine are discussed.

## Background

Exome Sequencing (ES) allows simultaneous screening for variants in the coding portion of all genes present in a patient’s genome. Over the last few years, ES has aided in the elucidation of the genetic basis of multiple genetic syndromes (for a review of some examples see Ku et al. [1]). The relatively low cost of ES and its’ high diagnostic yield have stimulated discussion about its promising role in clinic [2-4]. However, despite the unprecedented success of ES as a research tool, its utilization as a genetic screening test in clinic remains largely prohibitive due to challenges associated with consent, incidental findings, and the management of the massive amounts of data generated (see “Challenges of integrating ES in clinic” subsection). Furthermore, in many families there is a single affected individual available, which adds further complexity to the analysis of the results [5], unless the genetic variant responsible for the disease is not present in the parents.

### Challenges of integrating ES in clinic

Adapted from Trakadis [6], published with the permission of author.

1. **Meaningful patient informed-consent may not be feasible**

• *Possibility of incidental findings,*

• *Multiple findings of uncertain clinical significance,*

• *Multiple issues to discuss leading to prohibitive requirements in time & resources*

2. **Potential emotional distress over disease risk even among healthy individuals**

3. **Genomic information is a powerful personal identifier**

• *Raising concerns about privacy, confidentiality, genetic discrimination*

4. **Very large amounts of genetic information generated**

• *Limited number of clinical geneticists for data interpretation and clinical care*

• *Substantial time and cost for data analysis and genetic counselling*

• *Dynamic/evolving nature of the interpretation as new knowledge is gained*

• *Duty to re-contact patients as knowledge changes over time*

To address these challenges, variant prioritization using bioinformatic tools (e.g. Berg et al. [7]; Berg et al. [8]) and practice guidelines/recommendations (e.g. Christenhusz et al. [9]; ACMG Policy statement on Genomic Sequencing, May 2012 [10,11]) have been suggested. These approaches, however, do not adequately address all the challenges summarized in the “Challenges of integrating ES in clinic” subsection (e.g. incidental findings, findings of uncertain clinical significance, risk for genetic discrimination, requirements in time & resources). Moreover, they are limited by the efficiency of the bioinformatic tools to accurately predict the clinical impact of different variants [12,13]. At present, different tools often lead to opposite predictions about the functional impact of the same variant [14]. Nonetheless, the ability of ES to facilitate diagnosis and inform therapy will likely lead to its premature introduction in clinic using an approach similar to the one followed for chromosomal microarray [15-20].

In the light of rapid developments in genomic technologies, medical genetics is shifting from the present “phenotype-first” medical model to a “data-first” model, which leads to multiple complexities. An alternative phenotype-driven approach was recently put forward [6]. This approach, namely Individualized Mutation-weighed Phenotype On-line Search (I-MPOS), aims to address the above mentioned issues and facilitate widespread clinical utilization of ES. We hereby present PhenoVar, a software consistent with this phenotype-driven approach, and provide preliminary evidence of its potential benefits.

## Implementation

### PhenoVar and phenotype-driven analysis of exome data

**Figure 1:**
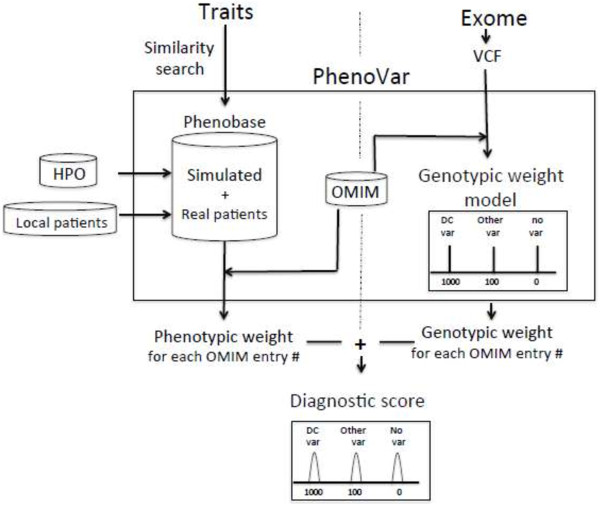
**Workflow of PhenoVar.** PhenoVar automatically prioritizes diagnoses for validation based on both the phenotypic and genomic information of a proband. It calculates a patient-specific diagnostic score for each OMIM entry with known molecular basis. The diagnostic score assigned to a given syndrome is the sum of its phenotypic and genotypic weight. For each syndrome listed in the HPO database the phenotypic weight is determined by calculating the similarity between the proband and the different patients available in a local database (Phenobase). Phenobase includes simulated patients using HPO and real patients (here denoted as “local patients”). The genotypic weight for each syndrome corresponds to the (predicted) pathogenicity of any variants present in the proband’s exome specifically in the gene(s) causing the respective syndrome. When no variation is found in these genes, the genotypic weight for that syndrome is automatically set to null value. Otherwise, the variants are sorted into known disease-causing variants (DC var) versus possibly pathogenic variants (other var) and assigned a different score. The *genotypic weight* and *phenotypic weight* described above are summed to obtain the *diagnostic score* for each syndrome. The different syndromes are then ranked according to their diagnostic score.

Figure 1 summarizes the overall workflow of PhenoVar. In brief, PhenoVar automatically prioritizes diagnoses for validation based on both the phenotypic and genomic information of a proband. It calculates a patient-specific diagnostic score for each OMIM entry (Online Mendelian Inheritance in Man; http://www.ncbi.nlm.nih.gov/omim) with known molecular basis. The diagnostic score assigned to a given syndrome is the sum of its phenotypic and genotypic weight.

#### Calculation of phenotypic weight

For each syndrome listed in the Human Phenotype Ontology (HPO; http://www.human-phenotype-ontology.org) the phenotypic weight is determined by calculating the similarity between the proband and the different (simulated) patients available in a local database, as described below.

In order to compare the phenotype of a patient with an unknown diagnosis to phenotypes corresponding to known genetic syndromes, we simulated a large number of sample patients, hereafter referred to as *simulated patients,* using HPO and OMIM databases. For each syndrome listed in the HPO database, twenty to twenty-five simulated patients were randomly generated using the phenotypic traits corresponding to that diagnosis and the information was stored in a local database (Phenobase). On average, a total of 5 traits corresponding to the respective disease were assigned to each simulated patient. The probability of each trait to be present in the phenotype of a given simulated patient was chosen to be proportional to the prevalence of that trait in the respective disease, as available in the HPO database. Only simulated patients corresponding to a syndrome with a known molecular basis, according to the OMIM database, are considered in the subsequent steps of the analysis.

Each trait entered by the user for a given proband is analyzed by PhenoVar using the HPO ontology, a directed acyclic graph representing the relationships between the traits. For each node (trait) in the graph, previous (parent) nodes are more generic traits and forward (child) nodes are more accurate descriptions of the trait. This allows more flexibility in the terms used by the clinician, while still enabling recognition of similarity between the proband and each (simulated) patient in Phenobase. For a given syndrome, accurate and general hits are limited to one child or parent node, in relation with traits listed in HPO. The PhenoVar algorithm first compares the proband to all patients contained in Phenobase (currently including mostly simulated but also some real patients with known diagnoses) and calculates a *phenotypic similarity weight* for each patient in PhenoVar relative to the proband. For every syndrome (represented by different patients in Phenobase) the phenotypic similarity weights are summed and then averaged to obtain the final *phenotypic weight for that syndrome*. A higher weight correlates with a higher likelihood that the proband is affected by this syndrome, based on phenotype only.

The following formula summarizes the details of this process:

(1) $$\frac{\sum_{i=1}^{\mathrm{nbPatient}}\exp\left(\mathrm{nbHi}t_{i}\times \mathrm{KH}+\mathrm{nbAccurateHi}t_{i}\times \mathrm{KA}+\mathrm{nbGeneralHi}t_{i}\times \mathrm{KG}-\mathrm{nbMis}s_{i}\times \mathrm{KP}\right)}{\mathrm{nbPatient}}$$

Where *nbPatient* is the number of simulated patients with the same syndrome; *nbHit*_*i*_ is the number of traits shared between the *i*^th^ simulated patient and the patient; *nbAccurateHit*_*i*_ is the number of traits in the patient that correspond to more accurate versions of a trait in the *i*^th^ simulated patient. *nbGeneralHit*_*i*_ is the number of traits in the patient that correspond to more general versions of a trait in the *i*^th^ simulated patient; *nbMiss*_*i*_ is the number of patient traits not matched with the *i*^th^ simulated patient; KH, KA, KG and KP are constant parameters which were determined based on an independent cohort of test patients (KH, KA and KG =2, KP = 1).

#### Calculation of genotypic weight

The genotypic weight for each syndrome corresponds to the (predicted) pathogenicity of any variants present in the proband’s exome specifically in the gene(s) causing the respective syndrome. Hence, the genotypic weight is generated in parallel for each syndrome using the proband’s exome VCF (Variant Call Format) data file. When no variation is found in these genes, the genotypic weight for that syndrome is automatically set to null value. Predetermined values (weight) are assigned to each variation in the VCF file, according to SNPEff annotation or known disease-causing status (ClinVar, HGMD).

Two different models are used in the present paper. In the first model all filtered variations are assigned by PhenoVar the same weight (arbitrary value of 1000), irrespective of zygosity. The final genotypic weight for each syndrome corresponds to the greatest variation weight across all genes causing the disorder. When no variation is found in these genes, the weight is automatically set to null value.

The only difference in the second model is that the variants are filtered and sorted in two groups: known disease-causing variants (group 1) versus possibly pathogenic variants (group 2). The disease-causing variants listed in ClinVar and HGMD, as described above, are classified in group 1, while the genetic variations with moderate or high functional impact based on SNPEff predictions (i.e. frameshift, missense, non-sense and splice sites) are classified in group 2. In this model, variants in groups 1 and 2 are arbitrary assigned a weight of 1000 and 100, respectively, and the remaining variants a null value.

#### Phenotype and genotype score integration, ranking of possible diagnoses and filter for incidental findings

The *genotypic weight* and *phenotypic weight* described above are summed to obtain the *diagnostic score* for each syndrome. The different syndromes are then ranked according to their diagnostic score. The syndrome with the highest diagnostic score represents PhenoVar’s prediction of the most likely diagnosis. An option to filter the ranked syndromes based on the suspected mode of inheritance is also available. Finally, using an empirically determined phenotypic threshold, disorders unrelated to the proband’s phenotype were filtered out.

To determine this phenotypic threshold (cut-off: 0.9705), simulated patients whose exome VCF files were modified to include a pathogenic variant corresponding to their diagnoses but also an incidental finding were used.

### Test-patients selection and preparation of variants files

Ten test-patients with different polymalformative genetic syndromes were randomly selected from previously published case reports (patients 1a to 10a in Table 1). For each patient selected, the mutation, phenotypic data, and genetic diagnosis were available in the published manuscript. Next, the VCF files of 10 control exomes were obtained from the National Institute of Environmental Health Sciences (NIEHS) Environmental Genome Project (EGP) (http://evs.gs.washington.edu). These control exome VCF files were modified so that each of them subsequently included the disease causing variant corresponding to one of the test patients (1a to 10a) in Table 1.

**Table 1:** Characteristics of the test patients selected from the literature

**Patient identification**	**Phenotype search traits (Patient reference)**	**Gene**	**Mutation**	**Correct diagnosis (OMIM)**
1a	Holoprosencephaly	*SIX3*	*c.977G > C* p. Arg257Pro	Holoprosencephaly-2 (157170)
	Microphthalmos			
	Iris coloboma			
	(Wallis et al.) [21]			
2a	Preaxial polydactyly	*NEK1*	c.379C > T p.Arg127Ter	Short rib-polydactyly syndrome, type II (263520)
	Median cleft lip and palate			
	Short ribs			
	(Thiel et al.) [22]			
3a	Cutaneous finger syndactyly	*SLC26A2*	c.1984 T > A p.Cys653Ser	Epiphyseal dysplasia, multiple, 4 (226900)
	Patellar dislocation			
	Scoliosis			
	(Makitie et al.) [23]			
4a	Polymicrogyria	*GPR56*	c.1036 T > A p. Cys346Ser	Polymicrogyria, bilateral frontoparietal (606854)
	Seizures			
	Microcephaly			
	(Piao et al.) [24]			
5a	Synophrys	*RAD21*	c.1127C > G p.Pro376Arg	Cornelia de Lange syndrome 4 (614701)
	Microcephaly			
	Tetralogy of Fallot			
	(Deardorff et al.) [25]			
6a	Micromelia	*COL2A1*	c.4172A > G p.Tyr1391Cys	Platyspondylic lethal skeletal dysplasia, Torrance type (151210)
	Radial bowing			
	Pulmonary hypoplasia			
	(Nishimura et al.) [26]			
7a	Generalized myoclonic seizures	*EHMT1*	c.3409C > T p. Arg1137Ter	Kleefstra syndrome/Chromosome 9q34.3 deletion syndrome (610253)
	Global developmental delay			
	Short stature			
	(Kleefstra et al.) [27]			
8a	Anophthalmia	*STRA6*	c.878C > T p.Pro293Leu	Microphthalmia, syndromic 9 (601186)
	Pulmonic stenosis			
	Blepharophimosis			
	(Pasutto et al.) [28]			
9a	Oligohydramnios	*RBM10*	c.1235G > A p. Trp412Ter	TARP syndrome (311900)
	Cleft palate			
	Defect in the atrial septum			
	(Johnston et al.) [29]			
10a	Hyperventilation	*TCF4*	c.1727G > A p.Arg576Gln	Pitt-Hopkins syndrome (610954)
	Postnatal microcephaly			
	Seizures			
	(Amiel et al.) [30]			
1b	Limb shortening	*WNT7A*	*c.1179C > T p.Arg292Cys*	*Ulna and fibula absence of with severe limb deficiency (276820* ***)***
	Aplasia/hypoplasia of the fibula			
	Aplasia/hypoplasia of the ulna			
	(Woods et al.) [31]			
2b	Synostosis of carpals/tarsals	*NOG*	c.104C > G p.Pro35Arg	Tarsal-carpal coalition syndrome (186570)
	Proximal symphalangism			
	Radial head subluxation			
	(Dixon et al.) [32]			
3b	Adrenal hypoplasia	*WNT4*	c.341C > T p.Ala114Val	Serkal syndrome or sex reversal, female, with dysgenesis of kidneys, adrenals, and lungs (611812)
	Intrauterine growth retardation			
	Renal agenesis			
	(Mandel et al.) [33]			
4b	Anal atresia	*GLI3*	c.2188_2207del	Pallister-Hall syndrome (146510)
	Central polydactyly (hands)			
	Short thumb			
	(Killoran et al.) [34]			
5b	Global developmental delay	*SC5DL*	c.86G > A p.Arg29Gln	Lathosterolosis **(**607330)
	Postaxial polydactyly of foot			
	Toe syndactyly			
	(Brunetti-Pierri et al.) [35]			
6b	Central polydactyly (feet)	*RAB23*	c.434 T > A p.Leu145Ter	Carpenter syndrome (201000)
	Craniosynostosis			
	Finger syndactyly			
	(Jenkins et al.) [36]			
7b	Cleft palate	*DHCR24*	c.571G > A p.Glu191Lys	Desmosterolosis (602398)
	Short stature			
	Aplasia cutis congenita			
	(Waterham et al.) [37]			
8b	Generalized hypotonia	*NSD1*	c.1310C > G p.Ser437Ter	Sotos syndrome (117550)
	Macrocephaly			
	Overgrowth			
	(Kurotaki et al.) [38]			
9b	Holoprosencephaly	*DHCR7*	c.832-1G > C	Smith-Lemli-Opitz syndrome (270400)
	Median cleft lip and palate			
	Microcephaly			
	(Wright et al.) [39]			
10b	Short stature	*IHH*	c.137C > T p.Pro46Leu	Acrocapitofemoral dysplasia (607778)
	Limb shortening			
	Cone-shaped epiphysis			
	(Hellemans et al.) [40]			

Table 1 summarizes the characteristics of the test-patients selected from the literature. The first column lists the identification number assigned to each patient. The phenotypic traits selected by the medical geneticist “blinded” to the diagnoses and the reference articles are listed in the second column. The affected gene, exact mutation, and corresponding diagnosis for each test-patient are also included in this table.

Next, ten genetic syndromes whose phenotypic features had a documented prevalence in HPO were identified. Each of these syndromes was then searched in OMIM for previously published case reports and one patient representing each syndrome was selected from the literature (1b to 10b in Table 1). The ten original (unmodified) control exome-files were now modified so that each of them subsequently included the disease causing variant corresponding to one of the test-patients 1b to 10b in Table 1.

Each test patient was thus assigned a specific exome VCF file modified to include his/her disease causing variant. The resulting files were first annotated using SNPEff (version 2.0.5) for variation functional impact, and then for known disease-causing variants as classified in ClinVar database (clinical significance = “4”, probable pathogenic or “5”, pathogenic) and in the professional version of Human Gene Mutation Database (HGMD) (Disease mutation –“DM” variants). The files were further filtered to exclude non-disease-causing intronic or synonymous variants, as well as variations with >5% frequency listed in dbSNP (build 135).

A medical geneticist, “blinded” to the diagnoses of the test-patients, was provided with the clinical description of the twenty patients. Based on our previous experience PhenoVar performs best when three or more traits are used. Using terms in HPO, the geneticist selected for each case three traits that he perceived as significant and more specific. Subsequently, for each patient, he introduced the selected terms along with the respective modified exome VCF file in the web-based interface of PhenoVar. The results obtained were analyzed for the position of the correct diagnosis, by the members of the team aware of the diagnoses, to test the efficiency of PhenoVar.

Next, the medical geneticist, while still blinded to the diagnoses, was asked to select different keywords and ensure that 2–3 of the keywords selected were present in Phenobase and the analysis was repeated. Of note, the number of matching traits from the traits entered is evident after each analysis with the software.

### Incidental findings

For each test-patient, we reviewed genes known to be responsible for mendelian disorders which harboured previously reported diseases-causing variants (ClinVar, HGMD) or variants predicted to be likely pathogenic (non-sense, frameshift, consensus splice site). To report incidental findings, we focused mostly on the ACMG minimal list [11].

Moreover, the modified control exome VCF files mentioned above, which were further modified by introducing a known *BRCA1* pathogenic variant (incidental finding) in each case, were analyzed. Entering this VCF file in PhenoVar along with corresponding set of 3 phenotypic traits summarized for each case in Table 1 allowed for testing the phenotype filter for incidental findings.

### Analysis of real patients with unknown genetic disorders

To illustrate that PhenoVar can be used with data from real patients, four patients with multiples congenital anomalies, previously diagnosed via exome sequencing to have a known mendelian disorder, were used. With regards to exome sequencing, DNA libraries were prepared for each patient (TruSeq, Illumina), followed by target enrichment (Agilent SureSelect All Exon kit v4) and sequencing on a HiSeq2000 (Illumina) with 3 exomes per lane, giving an average coverage of ~100X. Analysis of sequencing data was done with the GATK v2 package as per the recommendations of the Broad Institute [41]. SnpEff 2.0.5 (GRCh37/hg19) was used for variant annotation and ClinVar database for identification of known pathogenic variants. The variants identified were filtered using a cut off of 1% for minor allele frequency (dbSNP database built 137 and local control exomes). From the remaining variants, the ones predicted to alter amino acid sequences or consensus splice sites junctions, which were determined to not be tolerated by Polyphen2 or SIFT software, were manually reviewed under the supervision of a medical geneticist (SL). After confirming the diagnosis, the data analysis was repeated for these patients using PhenoVar, as illustrated in Table 2.

**Table 2:** Four real patients analyzed by PhenoVar

**Patient ID**	**Phenotype search traits (Patient reference)**	**Gene**	**Mutation**	**Correct diagnosis (OMIM)**	**PhenoVar ranking**
A	Cleft palate	*SATB2*	c.1165C > T (p.Arg389Cys)	Cleft palate, isolated; cleft palate and mental retardation (119540)	1
	Congenital myopia				
	Global developmental delay				
	Micrognathia				
B	Cutis laxa	*NBAS*	c.5741G > A(p.Arg1914His)/c.682insT (p.Cys228Fs)	Short stature, optic nerve atrophy, and Pelger-Huet anomaly (614800)	2
	Hydrocephalus				
	Intellectual disability				
	Optic atrophy				
C	Abnormality of dental enamel	*JUP*	c.902A > G (p.Glu301Gly)/c.902A > G (p.Glu301Gly)	Naxos disease (601214)	3
	Generalized ichthyosis				
	Palmar hyperkeratosis				
	Plantar hyperkeratosis				
	Woolly hair				
D	Congenital cataract	*COL4A1*	c.3149G > A (p.Gly1050Glu)	Porencephaly, Familial (175780)	7
	Intellectual disability				
	Microcephaly				
	Seizures				

Table 2 summarizes four examples illustrating that Phenovar can be used with real patients data. The first column lists the identification letter assigned to each patient. The phenotypic traits used when running PhenoVar are listed in the second column. The next three columns denote the affected gene, exact mutation, and corresponding diagnosis (as determined after standard analysis of all the data, i.e. without using PhenoVar) for each patient. Finally, the last column indicates the ranking assigned by PhenoVar to the correct diagnosis.

## Results

Table 1 summarizes the information of the patients selected from the literature, including only the phenotypic traits selected by the medical geneticist blinded to their diagnoses. The genetic syndromes represented in Table 1 include both autosomal recessive and dominant conditions. Table 3 lists the number of variants identified in the exome corresponding to each test patient and the ranking of the correct diagnosis by PhenoVar. On average 3942 variants were obtained per filtered exome, of which 53 and 23 were included as disease-causing in HGMD and ClinVar, respectively. When PhenoVar ranked the possible diagnoses based only on the phenotypic traits entered, its efficiency appeared to be similar to that of Phenomizer or OMIM search engines (data not shown). When ranking the possible diagnoses solely based on phenotypic weight, PhenoVar predicted the correct diagnoses in three patients but did not rank the correct diagnosis as part of the top 20 possible diagnoses in 9 out of 20 patients (Table 3, Column 3).

**Table 3:** Diagnosis prediction for test-patients using PhenoVar

**Patient identification number**	**Number of variants ≤ 5%**	**Phenovar ranking (phenotypic weight only)**	**PhenoVar ranking (equal genotypic weight model)**	**Phenovar ranking (disease-causing genotypic weight)**	**Matched traits**
1a	3631	11	1	1	2
2a	3848	1	1	1	3
3a	3842	>200	37	7	1
4a	3841	84	11	3	2
5a*	4353	26	2	1	2
6a*	3913	30	3	2	2
7a	3850	>200	131	22	1
8a*	3819	1	1	1	3
9a	4519	2	1	1	2
10a	3799	2	1	1	3
1b	*3631*	3	1	1	2
2b	3848	6	4	4	2
3b	3842	100	3	1	1
4b	3841	4	1	1	2
5b	4353	3	1	1	3
6b	3913	136	8	2	1
7b	3850	11	1	1	2
8b	3819	156	17	1	1
9b	4519	22	2	1	2
10b	3799	1	1	1	3

The first column in this table lists the identification number assigned to each test-patient. The number of variants with global minor allele frequency (GMAF) of less than 5% present in the modified exome assigned to each patient is highlighted in the second column. The next three columns denote the position of the correct diagnosis for each patient, as ranked by Phenovar using some of its different options: first solely based on the selected phenotypic traits of the respective patient (third column); next, by integrating the phenotypic traits and variants present in the exome of the patient: while assigning the same weight to all variants (fourth column); and finally, by assigning a higher weight to mutations known or predicted to cause disease (fifth column). The last column indicates how many of the traits selected by the medical geneticist “blinded” to the correct diagnoses matched any traits in Phenobase.

*Mutation annotated incorrectly (please refer to discussion).

By including both the patient’s phenotype and exome data, PhenoVar’s efficiency improved significantly. When assigning the same weight to all variants in the exome (Table 3, Column 4), PhenoVar predicted the correct diagnosis in 10 out of 20 patients, while in 15 out of 20 the correct diagnosis was among the 4 highest ranked possible diagnoses. The correct diagnosis was not ranked in the top 20 diagnoses in only two patients.

When using PhenoVar’s option to automatically assign a higher weight to variants known, or bioinformatically predicted, to cause disease, PhenoVar’s diagnostic yield increased to 14/20, with the correct diagnosis ranking in the top 4 highest ranked diagnoses in 18/20 patients. More specifically, in the second set of patients (1b-10b) the diagnosis was successfully predicted by PhenoVar in 8/10 patients and ranked within the top 4 diagnoses in the other two patients (Table 3, Column 5). Optimization of the selected keywords to ensure that two or three traits from the ones used were present in Phenobase further improved the diagnostic yield: PhenoVar successfully identified the correct diagnosis in 17 out of 20 patients, while 20/20 were ranked in the top 3 (data not shown).

With regards to incidental findings, two findings unrelated to the presenting complaint were identified when manually analyzing the VCF files of the test-patients. One of the variants was previously reported to cause Lynch syndrome (*MLH3*) and the other to cause Renal cell carcinoma/MODY type 3 (*HNF1A*). These incidental findings were not identified when using PhenoVar’s cut-off phenotypic threshold. Furthermore, when repeating the Phenovar analysis for all patients using the VCF files which had been further modified to include a variant known to cause the *BRCA1* cancer syndrome, this incidental finding was not identified using our cut-off phenotypic threshold.

Table 2 demonstrates that PhenoVar can also be used with real patients’ data. All four patients’ diagnoses (previously identified by standard bioinformatics analysis of exome sequencing results) were ranked highly by PhenoVar. Moreover, the two incidental findings known to be present in the real patients (specifically, a disease-causing variant for pigmented nodular adrenocortical disease, OMIM 610475, and another one *in BRCA2*, found in patients B and D, respectively) were not found using our cut-off phenotypic threshold.

## Discussion

With conventional approaches multiple genetic tests are typically required before a molecular diagnosis is reached. This leads to increased cost and time delay. Widespread use of clinical ES could accelerate genetic diagnosis to an unprecedented scale at low cost.

PhenoVar prioritizes diagnoses (mendelian disorders whose molecular bases are known) for validation based on both the phenotypic and genomic information of a proband. It was shown to perform very well with a limited number of phenotypic traits being used (three traits). Our data demonstrate that, by taking into consideration both the patient’s phenotype and encrypted exome data, the correct diagnosis for patients with different clinical presentations was prioritized more efficiently than relying solely on the patient’s phenotype (as seen when comparing columns 3 and 5 in Table 3). This was true when using different variants databases (ClinVar or HGMD, data not shown) or, to a lesser but still significant degree, when assigning the same weight to all variants present in an exome. Selecting PhenoVar’s option to assign the same weight for all variations present in a patient’s exome, rather than prioritizing the known pathogenic variants, has an important advantage: it minimizes the impact that erroneous variant classification, as benign or pathogenic, has on the efficiency of the software.

Interestingly, PhenoVar performed equally well for the cases where the phenotypic keywords selected were not specific for the correct diagnosis. For instance, in the case of patient 3b, although the phenotype-based ranking was very poor (Table 3, Column 3), the final ranking by PhenoVar was not compromised (Table 3, Columns 4 and 5). This suggests that PhenoVar will be particularly helpful in the unfortunate occasions where an important clinical trait is missed during the genetic evaluation or when dealing with atypical presentations of known genetic syndromes. In turn, diagnosing more patients with atypical presentations of known genetic syndromes would potentially help to better define the spectrum of clinical characteristics of these conditions. Additional studies using real patient data, in collaboration with individuals who will be using this software, need to be prospectively performed to achieve this goal but also to further validate this analysis tool. Table 2 summarizes four examples of real patients whose diagnosis was facilitated using PhenoVar. This table illustrates that PhenoVar can be used with real patient data. However, a follow-up study using a large cohort of patients will be needed to evaluate how effective PhenoVar is in prioritizing the correct diagnosis in a real clinical setting.

One of PhenoVar’s major advantages it that it optimizes prioritization of possible diagnoses taking into consideration the patient’s exome data without requiring an increase in the bioinformatics human resources available in the clinical setting. This could potentially allow for a widespread use of ES in clinical practice, as a screening test for known mendelian conditions. Moreover, through the optional use of a cut-off phenotypic weight threshold, the clinician can focus the analysis on the genetic causes which can potentially explain the specific phenotype/medical-issue at hand, thus preventing the undesired discovery of incidental findings. As a result, the approach described simplifies pre-test counselling and informed consent for exome sequencing as a clinical screening tool. It does not contradict but rather complements the bining approach previously put forward [7,10]. Widespread usage of ES in clinic will help evaluate the significance of different variants, including their penetrance/expressivity. It will thus aid in the identification of appropriate genes to target for screening [42,43], as well as, help improve the interpretation of incidental findings of interest to the patient.

As illustrated by cases 5a, 6a, 8a (Table 3) correct gene annotation (e.g. correct exon/intro borders) is crucial for the software to run properly, albeit, this limitation is not specific to PhenoVar, as it affects similarly the currently standard ES data analysis. Moreover, to optimize the efficiency of the software, the HPO database and Phenobase need to be properly curated. For instance, including in HPO the prevalence of the phenotypic features for different genetic syndromes has a significant impact on the efficiency of the software, as illustrated by PhenoVar’s efficiency in patients 1b-10b (Table 3). Including more real patients in Phenobase will, in time, overcome this problem. Also, an option to explore the presence or absence of a given variant in affected/unaffected family members (based on simultaneous comparison of their encrypted genomes) could allow for adjusting the weight assigned to different variants and further improve PhenoVar’s efficiency. Finally, the VCF files in our study were filtered to include variations with >5% frequency listed in dbSNP (build 135). The filter was intentionally set higher than usual to illustrate the efficiency of PhenoVar. However, since most genetic conditions are rare (low carrier frequency), one could opt to use a filter with a lower threshold (e.g. 1%). This would filter out more benign variants and thus improve the efficiency of PhenoVar.

The proposed approach follows the existing “phenotype-first” medical model and allows for better prioritization of the genes to be tested in a clinical lab. It is particularly useful in phenotypes caused by multiple different genes (e.g. evaluation of global developmental delay). Recent studies have provided evidence for the high diagnostic yield of exome sequencing [44-46]. Using ES as a screening test can increase the diagnostic yield of a clinical evaluation in a cost-effective fashion and decrease the time to diagnosis [2-4,47,48]. If used properly, PhenoVar can help address many of the challenges associated with integrating genomic technologies into clinical practice (see “Challenges of integrating ES in clinic” subsection). It remains the responsibility of the physician to seek confirmatory clinical diagnostic test targeting the suspected diagnosis and, for the unresolved cases, to clinically prioritize testing using the whole spectrum of clinical genetic testing modalities available.

## Future directions

**Figure 2:**
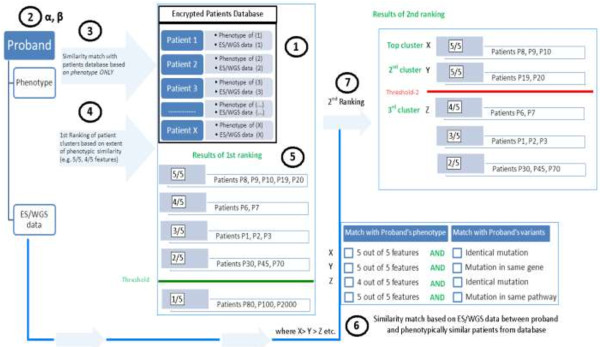
**Future directions about Phenovar or similar software using the I-MPOS approach and data from real patients.** A database containing phenotypic and encrypted genomic information of real patients with known or not-yet identified diagnoses can be made available **(1)**. A patient with an unknown diagnosis presents in clinic. His encrypted ES data are obtained and his phenotype is assessed **(2 α, β)**. The software automatically searches the “Encrypted Patients Database” using target patient’s assessed phenotype **(3)** thereby providing a first ranking of possible genetic conditions based on “phenotype weight” **(4), (5)**. For all patients in the database meeting a specific phenotype-similarity threshold in relation to the proband, the software will compare the changes present in their genomes against the ones present in the genome of the patient seen in clinic **(6)**. Matching the proband with the phenotypically similar subjects in the database based on similarity of their genetic changes (“mutation weight”) forms the basis of adjusting the first ranking to calculate the second ranking **(6), (7)**. Subjects sharing adequate phenotypic characteristics who also share a genetic variant cluster together. As a result, a given match is indicative of the possibility that the target subject shares the same genetic condition with the matched other subject(s). After the second ranking, the information about the shared phenotype and genotype of the patients clustering together is accessible and can aid in reaching the diagnosis. It should be noted that “phenotype” (steps 2–5) is not limited to clinical traits but also refers to other levels of phenotype, such as a metabolomic profile. Also, the word “mutation” (steps 6, 7) can refer to variants in more than one genetic loci which are simultaneously present in all matched patients allowing one to explore the possibility of gene-gene interaction.

In the future, Phenobase can be expanded to incorporate special databases containing phenotypic and genomic data of real patients [49,50], thus enabling encrypted on-line data sharing from consenting individuals. By including both patients with known and not-yet identified diagnoses in such interconnected encrypted databases, the method described above may have numerous benefits. First, when a large cohort of real patient data is made available in Phenobase, PhenoVar’s diagnostic yield for known syndromes would likely improve. This would be particularly true when atypical features are present. Secondly, continuously upgrading the database could allow the software to evolve and contribute in the identification of new syndromes as illustrated in Figure 2. In brief, a patient evaluated in clinic (target patient) could be automatically matched by the software with patients in the database based on phenotypic similarity. The software could then perform an automatic comparison of the exomes of the matched patients and that of the target patient. When the target patient’s exome file contains a variant shared by the phenotypically matched patients in the database whose diagnosis is known, his/her diagnosis could be inferred. In cases where the diagnosis of the phenotypically matched patients in the database is not yet known, an identical variant shared by these patients and the target patient could point to a candidate gene explaining their shared phenotype, a potentially newly described syndrome. This can be achieved while maintaining the genomic information of all participating patients in the database encrypted at all times.

Such an approach would also have implications about the delineation of heterogeneous complex genetic diseases with high heritability (e.g. schizophrenia [51-54]) into more homogenous endophenotypes based on subgroups of patients present in the database. Finally, the software could ultimately evolve to use, besides clinical traits, other levels of phenotypic information (e.g. metabolomic, transcriptomic, miRNomic data) when matching the target patient with patients in the database. The metabolome and/or other levels of phenotype, which can be accurately quantified and followed over time, constitute the downstream effect of unknown gene-gene or gene-environment interactions. If the metabolomic profiles of the patients in the database and the target patient were made available, such a software could integrate this information during the phenotype matching step. This approach would thus indirectly factor in differences in the genomic backgrounds and environmental exposures (both potentially influencing the pathogenic role of a specific shared variant). Hence, the *affected* patients with the shared variant would be prioritized, accounting for variations in penetrance and/or expressivity of different genetic conditions. As a result, this approach could facilitate screening, even in the newborn period, for genetic diseases whose biochemical phenotype (e.g. metabolomic profile) precedes the clinical presentation. Similarly, in time, such a tool could potentially be used at regular intervals in a patient’s lifetime through routine visits to a general clinic and facilitate the transition towards a more personalized practice of medicine [55].

## Conclusions

PhenoVar follows the existing “phenotype-first” medical model and facilitates the diagnostic approach by taking into consideration both the patient’s phenotype and all variations present in his exome, when ranking possible diagnoses (see Additional file 1). It is particularly useful in phenotypes caused by multiple different genes (e.g. evaluation of global developmental delay). Besides addressing many of the challenges associated with integrating genomic technologies into clinical practice, it can potentially provide in the future the infrastructure needed to further advance these tools safely and effectively.

## Availability and requirements

**Project name:** PhenoVar project.

**Project home page:**http://phenovar-dev.udes.genap.ca/.

**Operating system(s):** Platform independent.

**Programming language:** python.

**Other requirements:** No other requirement for the web bases version.

**License:** GNU GPL.

**Any restrictions to use by non-academics:** licence needed.

## Abbreviations

ES: Exome Sequencing; VCF: Variant Call Format; HGMD: Human Gene Mutation Database; HPO: Human Phenotype Ontology; I-MPOS: Individualized Mutation-weighed Phenotype On-line Search; OMIM: Online Mendelian Inheritance in Man.

## Competing interests

Patent application for the approach used is in process (by Y.J. Trakadis): to ensure that the I-MPOS paradigm can be properly implemented in a timely fashion. As long as the necessary academic collaborations for the programme are available, non-commercial applications of this method will remain free of charge.

Caroline Buote, Jean-François Therriault, Hugo Larochelle, Pierre- Étienne Jacques, and Sébastien Lévesque have no competing interests to declare.

## Authors’ contributions

Phenovar was designed by S. L., H. L. and Y. T. The experimental design of the study, interpretation of the results and the preparation of the first draft of the manuscript were performed by Y. T. and S. L. J-F T was responsible for programming; C. B. and P.E. J. for the bioinformatic analysis of the exome sequencing data of the real patients. C. B. also analysed the test-patients data. All authors read and approved the final manuscript.

## Pre-publication history

The pre-publication history for this paper can be accessed here:

http://www.biomedcentral.com/1755-8794/7/22/prepub

## Supplementary Material

Additional file 1 PhenoVar starting guide (Web version 1.0). Click here for file
