# Supplementary material for: PhenoVar: a phenotype-driven approach in clinical genomics for the diagnosis of polymalformative syndromes
Source: BMC Med Genomics. 2014 May 12;7:22. doi: 10.1186/1755-8794-7-22 (PMC4030287; doi:10.1186/1755-8794-7-22)

PhenoVar starting guide (Web version 1.0)

1. Accessing PhenoVar

- Access the homepage of the application at http://phenovar-dev.udes.genap.ca/. The homepage provides information about OMIM and HPO databases versions in use.
- To login, click on the icon "Phenotype form". You will be directed to the User authentification page. Enter the username and password that you have been provided, and click login. You will be directed to the phenotype form.

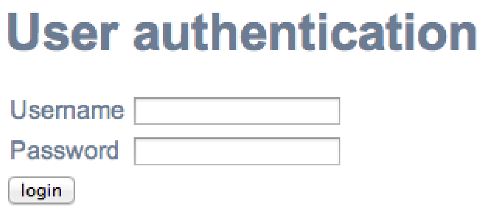

2. Retrieving an existing patient or a provided test patient

- To retrieve phenotypic and genotypic information of a previously saved patient, enter the identification number and click on the "Find patient" button as shown below. If a test patient was provided to you, you should have received the identification number with your username and password.

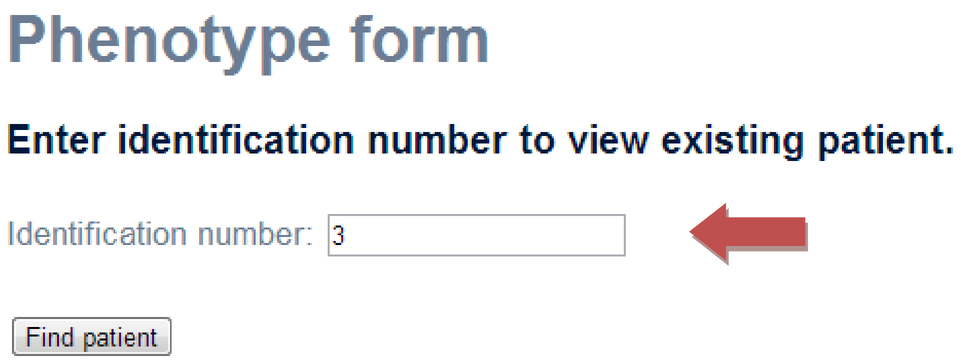

- The screen will then display basic patient information, the exome VCF (Variant Call Format) file in use, and the phenotypic description of the saved patient.

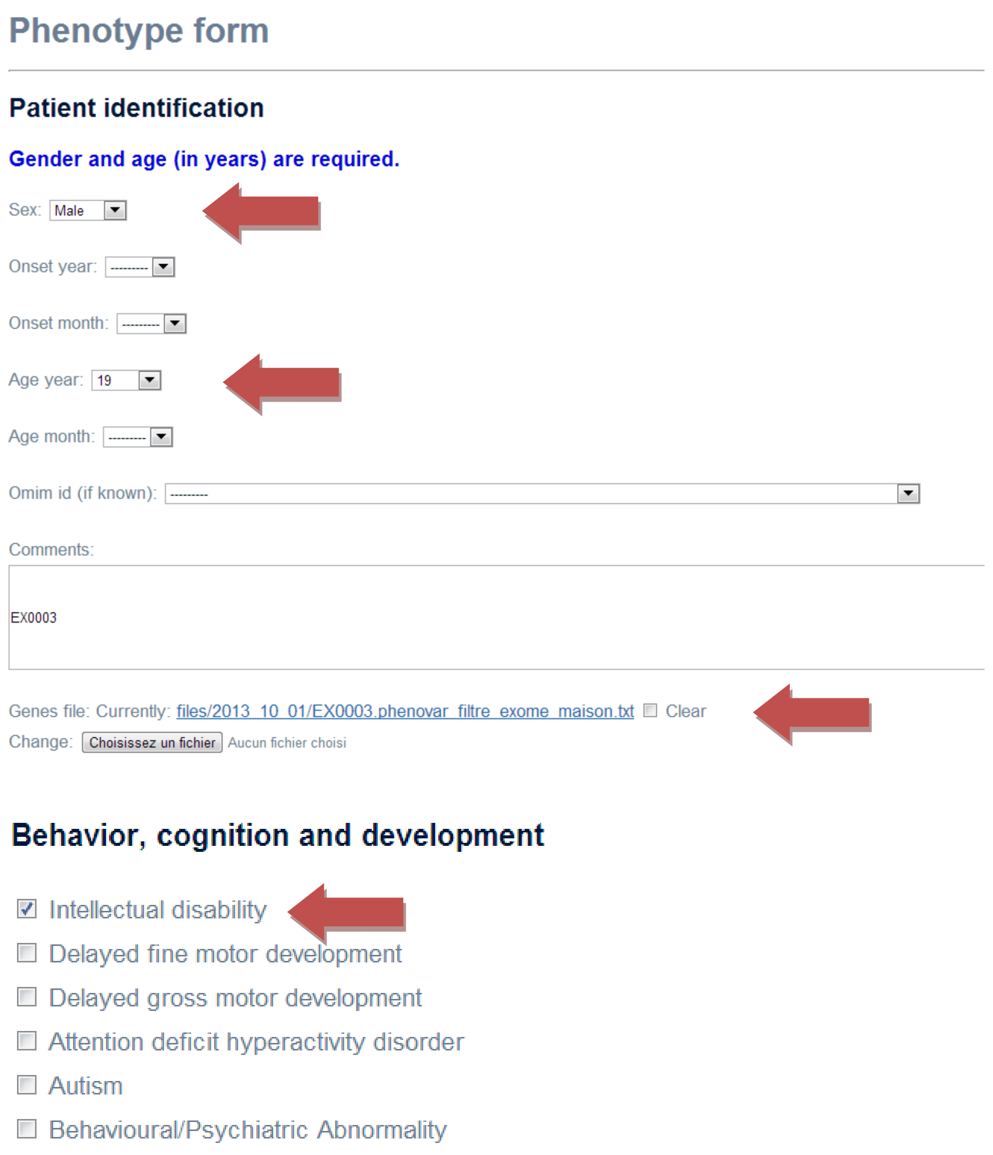

- Phenotype can be edited prior to submitting the phenotype form to the diagnostic tool. Use checkboxes or the search engine (at the bottom of the page).
- You can upload a new exome VCF file (“Change” field) or remove the existing file from the server (“Clear” checkbox). Please see the required format in the next section.
- You can then save edits, continue by adding a new patient or proceed directly to diagnostic support for the current patient.

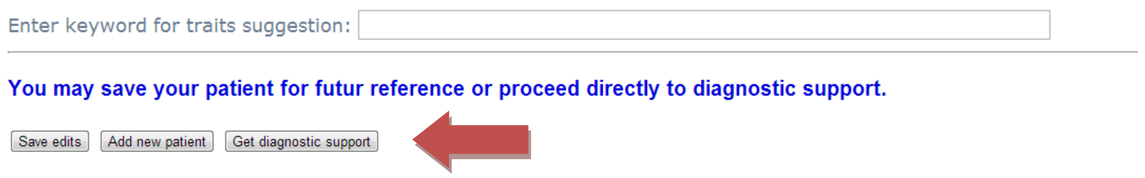

3. Entering a new patient

- **Patient general information:** enter the general patient information in the "Phenotype form", as shown above. Gender and age are required to save the patient information or to use the diagnostic tool. The optional field "Omim id" is used to indicate a known diagnosis. These data will be used to improve the performance of PhenoVar. Comments field is optional as well but can be used to add non-confidential information about the patient for future reference (ex: genetic tests, suspected disease inheritance).

- **Exome VCF file:** upload the patient exome VCF file using the field "Genes file". Variations annotations must have been performed with SnpEff. If your VCF file was filtered for known mutations in databases (ClinVar, HGMD, etc), please ensure that the term "Patho_5" appears in the "FILTER" column of the VCF file to identify those mutations. Variations flagged as "Patho_5" are given a higher genotypic score.

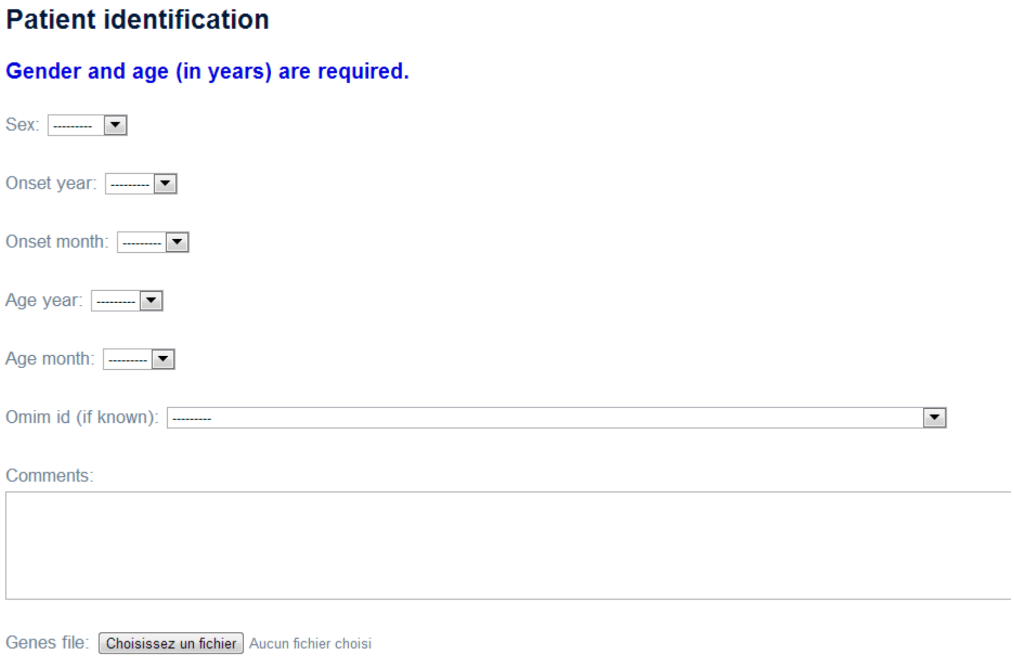

- **Phenotype description:** enter the phenotype description of the patient. Frequently used traits are listed under different subheadings (growth, craniofacial, etc). Use checkboxes to select them as depicted above. If you cannot find a trait in those listed or prefer to search for traits, you can enter free text in the search engine at the bottom of the page ("Enter keyword for traits suggestion"). Click on the trait to make it appear in the phenotype description. This trait finder widget is spelling sensitive and does not auto-correct.

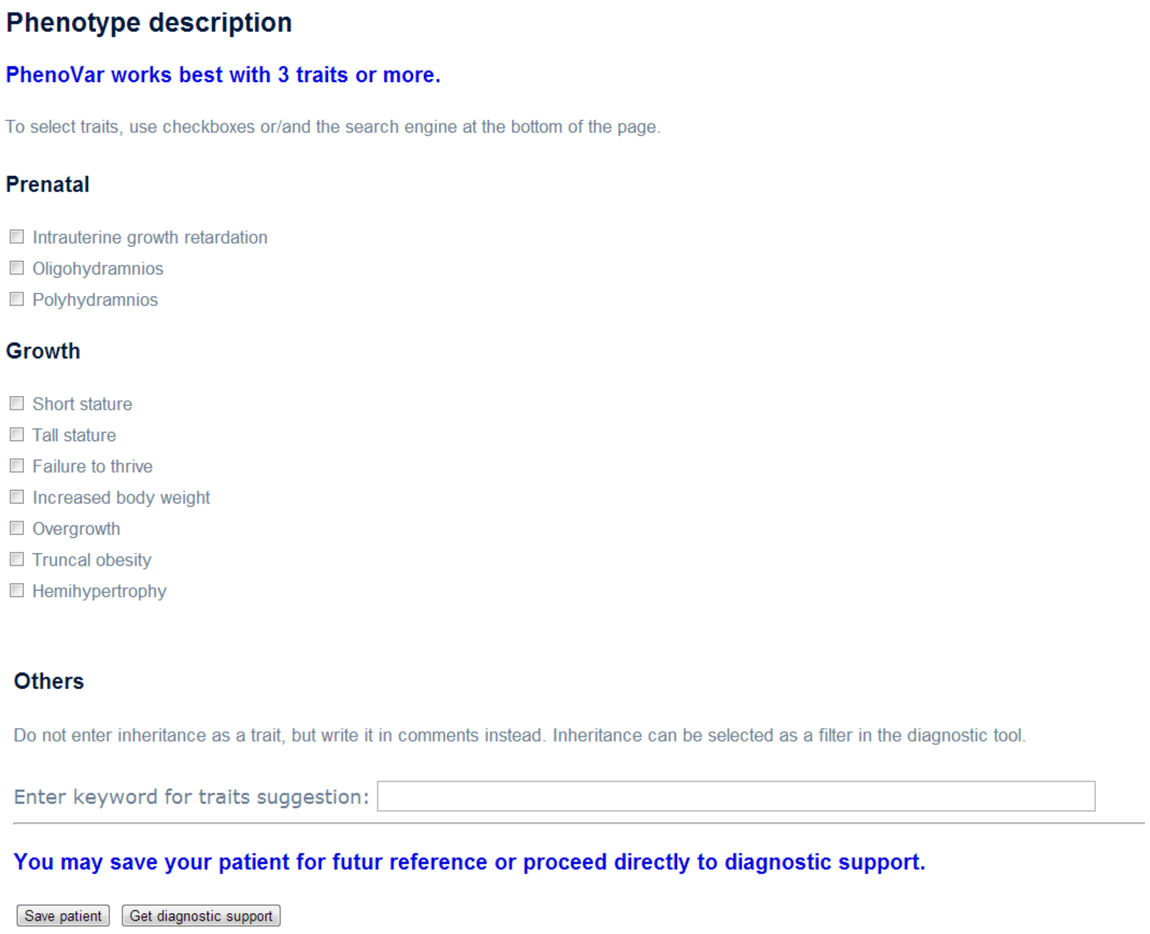

- You can save the patient phenotypic and genotypic information by clicking the “Save patient” button at the bottom of the page. **Warning**: saved patients cannot be deleted. You must ask the administrator to remove it. However, exome file can be removed any time.
- Click on “Get diagnostic support” to proceed to diagnostic tool.

4. Using the diagnostic tool

Phenotypic traits of the patient will appeared on the top of the page. Those traits have been used by PhenoVar to perform the similarity search against other patients of Phenobase. Here is a brief description of the important field on the page:

- **"Inheritance" drop down menu:** can be used to filter results per inheritance.
- **Phenotypic threshold:** allow to display only OMIM disorders that are phenotypically related to your patient and mask incidental findings. By default, the phenotypic threshold is on.
- **"Send feedback" button:** it is used to send user feedback entered in the disease score table.
- **Disease score table:** list of diagnostic predictions (OMIM entries) based on the phenotype and the exome data of your patient. Diseases are listed in order from the highest diagnostic score to the lowest. Phenotypic and genotypic scores are provided. Each disease has buttons to show the matching traits and matching variations (except if phenotype or genotype is missing). The user can enter feedback on PhenoVar predictions using the column located at the right side of the disease table. For a given suggested OMIM entries, the user can indicate if it constitutes a good prediction based on the patient's phenotypic description, or not. Feedbacks are optional but will be used in the future to improve the diagnostic predictions of the software. Once you have entered your feedback, send it by pressing the "Send feedback" button on top of the disease score table.

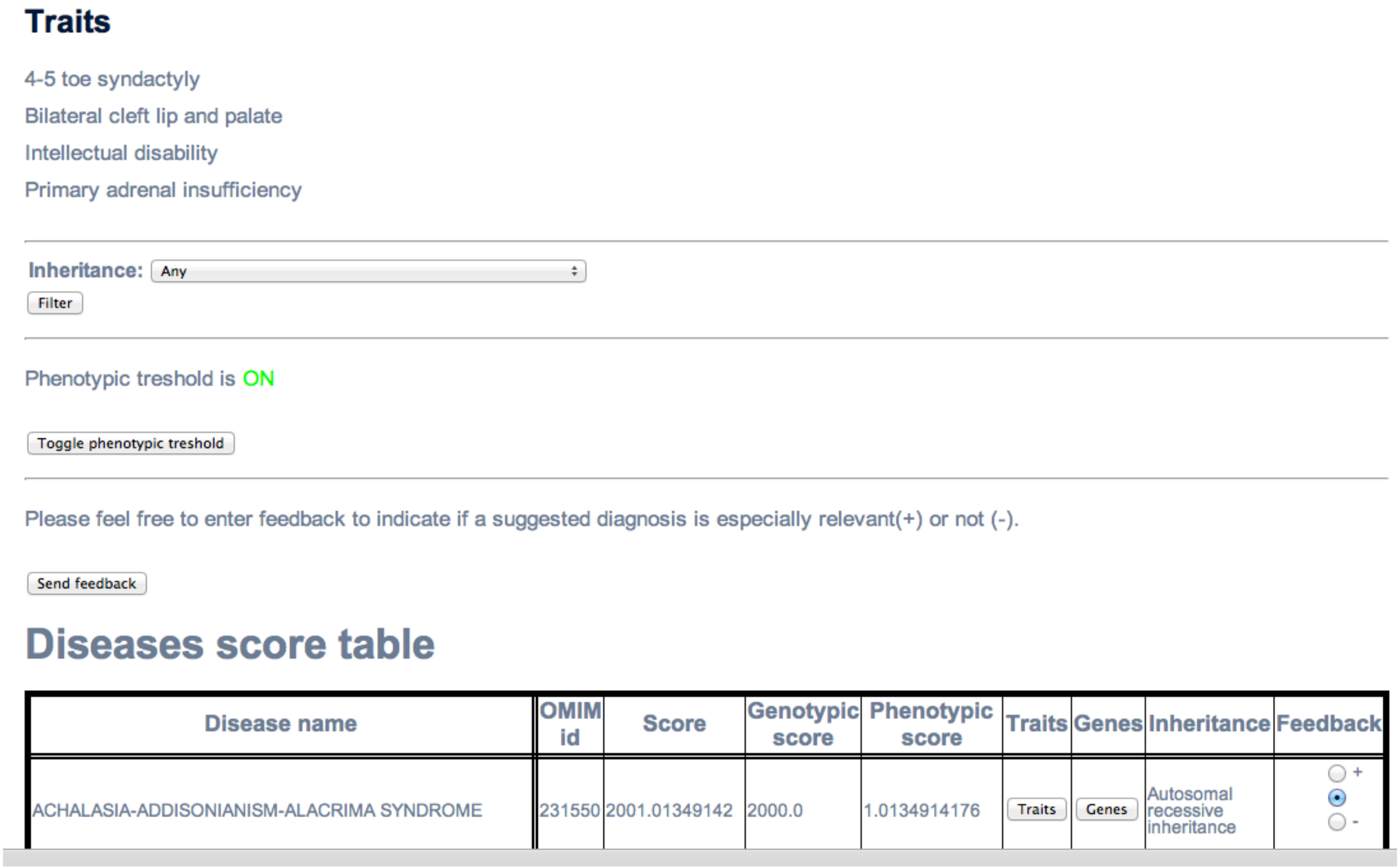

Traits

4-5 toe syndactyly
Bilateral cleft lip and palate
Intellectual disability
Primary adrenal insufficiency

Inheritance: Any
Filter

Phenotypic treshold is ON

Toggle phenotypic treshold

Please feel free to enter feedback to indicate if a suggested diagnosis is especially relevant(+) or not (-).

Send feedback

Diseases score table

Disease name	OMIM id	Score	Genotypic score	Phenotypic score	Traits	Genes	Inheritance	Feedback
ACHALASIA-ADDISONIANISM-ALACRIMA SYNDROME	231550	2001.01349142	2000.0	1.0134914176	Traits	Genes	Autosomal recessive inheritance	+ / -

- You can use the back button of your web browser to come back to the “Phenotype form” page, modify the phenotype traits and perform again the analysis.
- To begin to work on a new patient, click on Phenotype form icon on the top of the page.

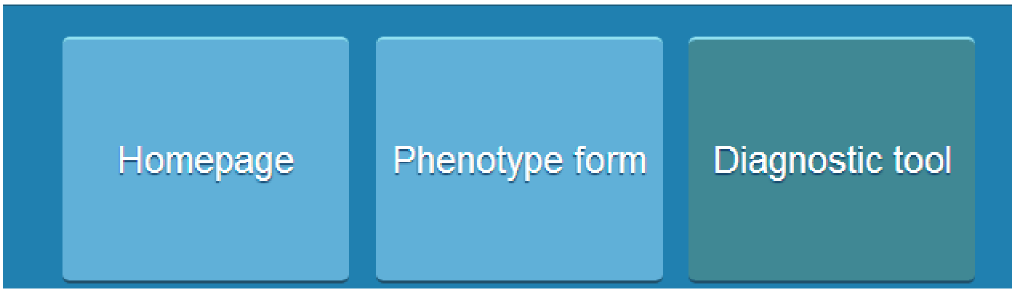

Supplement: Additional file 1 — PhenoVar starting guide (Web version 1.0). [file 1755-8794-7-22-S1.pdf]
